# Post-operative liver metastasis from gastric cancer with HER2-positive areas newly identified upon re-evaluation, leading to a favorable response to trastuzumab-based therapy: A case report

**DOI:** 10.3892/mi.2026.327

**Published:** 2026-06-11

**Authors:** Shinsuke Usui, Kenichi Iwasaki, Ryota Sakon, Ryuichi Wada, Tatsushi Suwa

**Affiliations:** 1 Department of Surgery, Kashiwa Kousei General Hospital, Kashiwa, Chiba 277-8551, Japan; 2 Department of Diagnostic Pathology, Kashiwa Kousei General Hospital, Kashiwa, Chiba 277-8551, Japan

**Keywords:** gastric cancer, HER2 reassessment, liver metastasis, trastuzumab, chemotherapy, biomarker

## Abstract

Recent advances in molecular targeted agents and immune checkpoint inhibitors have markedly improved outcomes in patients with unresectable or recurrent advanced-stage gastric cancer. As treatment options continue to expand, biomarker testing has become essential for determining first-line treatment strategies. Among these, human epidermal growth factor receptor 2 (HER2) plays a key role in treatment selection; however, the intratumoral heterogeneity of HER2 expression in gastric cancer has long been recognized and may influence therapeutic decision-making. The present study describes the case of a patient who was initially diagnosed as having a HER2 score of 0 on the surgical specimen, but was later found to have a HER2 score of 2+ with FISH positivity upon reassessment, subsequently demonstrating a favorable response to trastuzumab-based therapy. The patient was a 79-year-old man with a type 3 lesion at the cardia, and enlarged lymph nodes adjacent to the primary tumor. The pre-operative diagnosis was cT4aN1M0, stage III. He underwent total gastrectomy. At 27 months post-surgery, MRI revealed a 21-mm metastatic lesion in liver segment 8. Given the initial HER2 score of 0, nivolumab plus the oxaliplatin + leucovorin + 5-fluorouracil regimen (mFOLFOX6) was initiated as first-line systemic therapy. However, as tumor markers continued to increase after two cycles, HER2 was re-evaluated using a different section from the resected specimen, which revealed a score of 2+ with FISH positivity. Therefore, the treatment was switched to trastuzumab plus mFOLFOX6. Following the regimen change, tumor markers decreased, and a CT scan demonstrated the shrinkage of the liver metastasis to 13 mm. Chemotherapy remains ongoing 7 months following the initiation trastuzumab treatment. On the whole, the present case report suggests that the reassessment of HER2 status may help identify patients who may benefit from HER2-targeted therapy, and may ultimately improve the therapeutic efficacy of chemotherapy.

## Introduction

Trastuzumab was the first molecular-targeted agent approved in Japan for the treatment of unresectable or recurrent advanced-stage gastric cancer, with its efficacy established by the trastuzumab for gastric cancer (ToGA) trial (1). Since then, the development of molecular-targeted therapies and immune checkpoint inhibitors has continued to advance, leading to the approval of several new agents, including ramucirumab [based on the RAINBOW trial (2) and REGARD trial (3)], nivolumab [ATTRACTION-2 trial (4)], pembrolizumab [KEYNOTE-158 trial (5)], trastuzumab deruxtecan [DESTINY-Gastric01 trial (6)], and more recently, the zolbetuximab [SPOTLIGHT trial (7) and GLOW trial (8)]. As a result, therapeutic options for advanced-stage gastric cancer have steadily expanded.

According to the ‘Guidelines for Biomarker Testing, 2nd Edition’ published by the Japanese Gastric Cancer Association (9), the evaluation of human epidermal growth factor receptor 2 (HER2), programmed death-ligand 1 (PD-L1), microsatellite instability/mismatch repair and CLDN18.2 is recommended as essential biomarkers. Among these, HER2 testing is positioned as the starting point of the biomarker-based treatment cascade. HER2 positivity is observed in ~15-20% of all gastric cancers and is more frequently observed in differentiated adenocarcinomas, proximal gastric cancers and gastroeophageal junction (GEJ) cancers (10). However, the intratumoral heterogeneity of HER2 expression remains a clinical challenge, with factors, such as sampling location, specimen processing and interobserver variability in pathological assessment known to influence diagnostic accuracy. The present study reports a case in which a patient diagnosed as having a HER2 score of 0 on the surgical specimen developed liver metastasis 27 months following surgery. Although treatment with nivolumab plus the oxaliplatin + leucovorin + 5-fluorouracil regimen (mFOLFOX6) was initiated, the response was considered insufficient after two cycles. At that time, HER2 was re-evaluated using a different section of the resected specimen, which revealed a score of 2+ with fluorescence *in situ* hybridization (FISH) positivity. This finding prompted a switch to trastuzumab plus mFOLFOX6, resulting in tumor shrinkage.

## Case report

A 79-year-old man presented to Kashiwa Kousei General Hospital (Kashiwa, Japan) with epigastric pain. An upper gastrointestinal endoscopy revealed a type 3 lesion at the cardia (Fig. 1A), and a biopsy confirmed a diagnosis of moderately differentiated adenocarcinoma (tub2). The levels of serum tumor markers were elevated, with the levels of carcinoembryonic antigen (CEA) at 27.7 ng/ml and carbohydrate antigen 19-9 (CA19-9) at 2,164 U/ml. A contrast-enhanced abdominal computed tomography (CT) scan demonstrated gastric wall thickening with central depression at the cardia and multiple enlarged lymph nodes adjacent to the primary tumor (Fig. 1B). Lymph node enlargement was confined to the regional lymph nodes, with no para-aortic lymphadenopathy and no evidence of distant metastasis to the liver, lungs, or other organs.

Based on the 15th edition of the Japanese Classification of Gastric Carcinoma (11), the clinical diagnosis was cT4aN1M0, stage III. The patient underwent robot-assisted total gastrectomy with D2 plus lower mediastinal lymph node dissection and Roux-en-Y reconstruction. Due to esophageal invasion, the esophagus was transected 20 mm above the tumor, and esophagojejunostomy was performed within the lower mediastinum. The duration of the surgery was 511 min, and blood loss was 200 g. The post-operative course was uneventful, with no anastomotic leakage or pancreatic fistula; oral intake was initiated on post-operative day (POD) 6, and the patient was discharged on POD 20. A final pathological examination revealed well- to moderately differentiated adenocarcinoma, type 3, pT3N2M0, stage IIIA. The details are illustrated in Fig. 1C.

The levels of tumor markers normalized post-operatively, with the levels of CEA at 4.1 ng/ml and CA19-9 at 28 U/ml. Post-operatively, adjuvant chemotherapy with docetaxel plus S-1 at 60% of the standard dose was administered every 3 weeks for nine cycles, followed by S-1 monotherapy at a 60% dose.

At 12 months following surgery, gallbladder wall thickening was detected, and laparoscopic cholecystectomy was performed as malignancy could not be excluded. A pathological analysis revealed adenomyomatosis with chronic cholecystitis. Due to declining physical strength, chemotherapy was discontinued thereafter, and the patient was followed without treatment.

From 18 months post-operatively, the levels of CEA and CA19-9 gradually increased. At 25 months, the CEA level was 15.5 ng/ml and the CA19-9 level was 441 U/ml. A contrast-enhanced CT scan revealed a newly appeared 15-mm low-density area in segment 8 of the liver, raising suspicion of liver metastasis (Fig. 2A). PET-CT was subsequently performed, but no abnormal uptake was observed, and a definitive diagnosis of liver metastasis could not be established. At 27 months, the CEA level increased to 20.6 ng/ml and the CA19-9 level to 572 U/ml. An MRI revealed an enlarged 21x19 mm round lesion in segment 8 of the liver, exhibiting low signal intensity on T1-weighted imaging and mildly high signal on T2-weighted imaging, leading to a diagnosis of liver metastasis from gastric cancer (Fig. 2B). Metastatic lesions were not detected in any organs other than the liver.

The biomarkers of the carcinoma cells were analyzed by immunohistochemistry (IHC) using 4-µm-thick paraffin sections, composed of well-differentiated adenocarcinoma (Fig. 3A, No. 1). The paraffin sections were stained with hematoxylin and eosin (H&E). The paraffin sections were deparaffinized, stained with hematoxylin (Muto Pure Chemicals) for 6.5 min at room temperature and eosin (Muto Pure Chemicals) for 1.5 min at room temperature. HER2 was immunostained by Ventana BenchMark GX (Roche Diagnostics) using PATHWAY anti-HER-2/neu (4B5) rabbit monoclonal primary antibody (cat. no. 518-107918; prediluted; Roche Diagnostics) and ultraView Universal DAB Detection kit (cat. no. 518-109431; Roche Diagnostics), containing DAB Inhibitor, HRP multimer (prediluted), DAB chromogen, DAB H_2_O_2_ and DAB Copper and counterstained with Hematoxylin II and Bluing reagent (Roche Diagnostics) according to the standard clinical protocol. The primary antibody was incubated 37˚C for 16 min. The H&E- and immune-stained sections were examined under a blight-field microscope Axioscope 5 (Zeiss). The expression of HER2 was scored according to Japanese Guidelines for HER2 Testing in Breast Cancer/Gastric Cancer, 2nd Edition (12). The expression of HER2 was not found in carcinoma cells, and the score was diagnosed as 0 (Fig. 3B). IHC of PD-L1 was immunostained by Autostainer Link 48 (Agilent Technologies Japan, Ltd., Tokyo, Japan) using PD-L1 IHC 28-8 pharmDx kit (cat. no. SK00521-5J; Agilent Technologies, Inc.), containing EnVision FLEX target retrieval solution low pH, peroxidase blocking reagent, monoclonal rabbit anti-PD-L1 (clone 28-8; prediluted), linker (anti-rabbit) and visualization reagent-HRP DAB+ substrate buffer, DAB+ chromogen, DAB enhancer and counterstained with EnVision FLEX hematoxylin (Agilent Technologies Japan, Ltd) according to the standard clinical protocol. The primary antibody was incubated at room temperature for 30 min. The combined positive score (CPS) of PD-L1 was calculated as the number of PD-L1-positive cells including tumor cells, lymphocytes and macrophages divided by the number of tumor cells x100. The CPS was estimated >5. Therefore, nivolumab (100%) plus mFOLFOX6 (60%) was initiated and administered every 2 weeks. After two cycles, the level of CEA exhibited a slight decrease from 28.6 ng/ml at the initiation of chemotherapy to 27.4 ng/ml, whereas the level of CA19-9 markedly increased from 881 to 1,312 U/ml. Although the assessment was made shortly following the initiation of chemotherapy, the response to nivolumab-based treatment was considered insufficient, prompting the re-evaluation of the HER2 status. Re-evaluation was performed on the section, which was located close to the section for the initial evaluation and composed of well-differentiated adenocarcinoma (Fig. 3A, No. 2). IHC of HER2 was performed by Ventana BenchMark GX (Roche Diagnostics) using prediluted primary antibody PATHWAY anti-HER-2/neu (4B5) rabbit monoclonal primary antibody (cat. no. 518-107918; prediluted; Roche Diagnostics) and ultraView Universal DAB Detection kit (cat. no. 518-109431; Roche Diagnostics) and counterstained with Hematoxylin II and Bluing Reagent (Roche Diagnostics) according to the standard clinical protocol. The primary antibody was incubated 37˚C for 16 min. The moderate membranous expression of HER2 was noted in ~10% of carcinoma cells, and the expression of HER2 was scored as 2+ (Fig. 3C). FISH was performed using the PathVysion HER2 DNA Probe kit (Abott Japan LLC) according with standard clinical protocol, and the numbers of HER2 and CEP17 were counted under a fluorescence microscope Olympus BX-63 (EVIDENT; Olympus Corp.) according to Japanese Guidelines for HER2 Testing in Breast Cancer/Gastric Cancer, 2nd Edition (12). The HER2/CEP17 ratio was calculated as 3.9, indicating the amplification of the ERBB2 gene. Based on these findings, the patient received three courses of a nivolumab-containing regimen, after which the treatment was switched to trastuzumab (100%) plus mFOLFOX6 (60%) regimen administered every 3 weeks. After one cycle, the levels of tumor markers decreased (CEA, 19.3 ng/ml; and CA19-9, 1,061 U/ml); after two cycles, these levels further declined (CEA, 12.9 ng/ml; and CA19-9, 569 U/ml). The overall trend of CEA and CA19-9 throughout the clinical course is presented in Fig. 4. A contrast-enhanced CT scan at 31 months revealed that the segment 8 liver metastasis had decreased to 13 mm (Fig. 2C), representing a 38% reduction compared with the MRI performed at 27 months. Although this does not constitute a formal RECIST assessment as the measurements were obtained using different imaging modalities, the degree of shrinkage corresponds to a partial response. Due to a decline in physical strength, the mFOLFOX6 dose was reduced to 50% from the fifth cycle onward, with administration performed every 4 weeks. The disease remained dormant through the ninth cycle (37 months post-operatively), with the level of CEA at 17.1 ng/ml and that of CA19-9 at 544 U/ml. At the tenth cycle (38 months post-operatively), the levels of tumor markers again showed an upward trend (CEA, 24.1 ng/ml; CA19-9, 582 U/ml). A contrast-enhanced CT scan revealed that the metastatic lesion in liver segment 8 had enlarged to 23 mm; therefore, second-line therapy with ramucirumab (100%) plus paclitaxel (60%) was administered every 2 weeks at 39 months post-operatively, and treatment is ongoing.

## Discussion

Advances in systemic chemotherapy for unresectable or recurrent advanced-stage gastric cancer have highlighted the increasing importance of biomarker testing. In particular, biomarkers that function as companion diagnostics, such as HER2, PD-L1 and CLDN18.2 play a critical role in determining eligibility for specific therapeutic regimens. According to the additional analyses of the ToGA trial (10), the overall HER2-positive rate in gastric and GEJ cancers was 22.1%, with a notably higher rate of 32.2% in GEJ cancers. Furthermore, HER2-positivity was significantly more common in intestinal-type tumors than in diffuse-type tumors (31.8 vs. 6.1%).

HER2 heterogeneity has long been recognized as a clinically relevant issue. A nationwide Japanese survey evaluating HER2 and PD-L1 testing revealed substantial inter-institutional variability in HER2 scoring (13). According to this report, differences in tissue fixation methods and the time interval prior to fixation were identified as potential contributing factors. Notably, prolonged fixation was associated with an increased proportion of HER2 cases with a score of 0 or 1 and a decreased proportion of cases with a score of 3. Similar variability was also reported for PD-L1 expression, suggesting that pre-analytical factors and institutional practices significantly influence biomarker assessment.

The extent to which HER2 expression can be detected upon retesting is of particular clinical interest. The GASTHER1 trial investigated the utility of repeat biopsies in initially HER2-negative advanced-stage gastric cancer (14). That study reported a rescued HER2-positive rate of 8.7% following re-biopsy of the primary tumor. Notably, the likelihood of conversion to HER2 positivity increased with higher initial IHC scores (0 → 6.7%, 1 → 15.4%, 2 → 25%) (14). These findings suggest that HER2 expression may evolve over time or differ across tumor sites. However, in Japan, repeated biomarker testing is not routinely reimbursed under the national health insurance system, limiting the feasibility of systematic retesting in clinical practice. As regards the case described herein, it was decided to re-evaluate the HER2 status using a different section of the gastrectomy specimen as the tumor was a differentiated adenocarcinoma located at the gastroesophageal junction, where HER2 positivity is more common, and as the patient exhibited early biochemical progression within 1 month of initiating nivolumab-based first-line therapy. The re-evaluated section was processed using the same fixation method and assessed by the same pathologist as in the initial evaluation.

The GASTHER1 trial also reported that among patients whose primary tumors were HER2-negative, the rescued HER2-positive rate was highest in liver metastases (17.2%), which was 5.88-fold higher than in other metastatic sites (14). This observation suggests that metastatic lesions may harbor higher HER2 expression than the primary tumor. In the case presented herein as well, it is possible that HER2 expression was higher in the liver metastasis than in the primary lesion. However, a needle biopsy for HER2 testing of the liver metastasis was not performed due to the risk of peritoneal dissemination and bleeding from the punctured liver.

Although no standardized treatment guidelines exist for hepatic metastases from gastric cancer, several clinical studies have explored the role of hepatectomy and perioperative chemotherapy (15,16). In the case described herein, following four cycles of trastuzumab plus mFOLFOX6, the liver metastasis had decreased in size and remained solitary in segment 8. Although hepatectomy was considered, partial resection of segment 8 is not minimally invasive even when performed laparoscopically. Given the advanced age of the patient and his declining physical strength, surgical intervention was deferred in favor of continued systemic therapy.

Chemotherapy in elderly patients requires careful consideration of the dosage and treatment intensity. The GO2 Phase 3 trial demonstrated that reduced-dose capecitabine + oxaliplatin (0.8x and 0.6x) was non-inferior to standard dosing in 514 elderly or frail patients with gastroesophageal cancer, supporting the use of dose-attenuated chemotherapy to maintain disease control while preserving QOL (17). In the case in the present study, chemotherapy was initiated at 60% of the standard dose, and from the fifth cycle onward, the dosing interval was extended to every 4 weeks to accommodate the reduced physical reserve of the patient. Despite these adjustments, the patient maintained disease stability for 6 months through the ninth cycle, achieving outcomes comparable to the median progression-free survival of 6.7 months reported in the ToGA trial, indicating that the reduced dosing was appropriate.

The present case report describes a case in which a tumor initially classified as HER2-negative was reclassified as HER20-positive upon re-evaluation of a different area of the same resected specimen, leading to a favorable response to trastuzumab-based chemotherapy. In gastric cancer, where the intratumoral heterogeneity of HER2 expression is frequently observed, repeat HER2 testing based on tumor location and histopathological features may help identify latent HER2-positive cases even among those initially judged to be negative. Such re-evaluation efforts have the potential to more accurately identify patients who may benefit from HER2-targeted therapy and ultimately improve the therapeutic efficacy of chemotherapy for unresectable or recurrent disease.

## Figures and Tables

**Figure 1:**
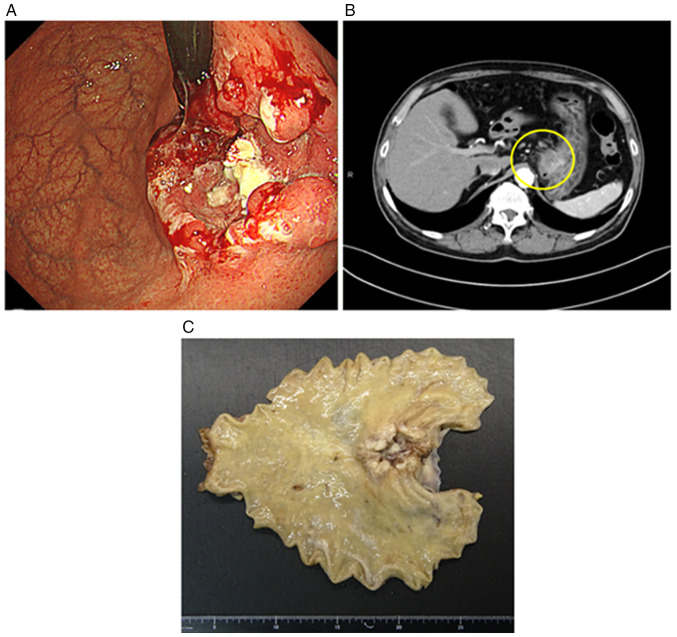
Upper gastrointestinal endoscopic findings, macroscopic finding of the resected stomach and pathological findings, and contrast-enhanced CT scan prior to surgery. (A) Upper gastrointestinal endoscopy illustrating a type 3 lesion at the cardia, accompanied by minimal esophageal involvement. (B) Contrast enhanced abdominal CT scan illustrating gastric wall thickening with central depression at the cardia and multiple enlarged lymph nodes adjacent to the primary tumor, which are highlighted within the yellow circle. (C) Macroscopic examination of the resected stomach demonstrating a type 3 tumor measuring 60x50 mm, and histopathological analysis revealed well- to moderately differentiated tubular adenocarcinoma, classified as pT3, pN2 (4/60), Ly1b, V1a, with negative proximal and distal margins (pPM0, pDM0). The biomarker analysis showed HER2 score of 0, pMMR, CLDN18.2-negative, and PD-L1 (28-8) CPS >5, as described in the text. CT, computed tomography.

**Figure 2:**
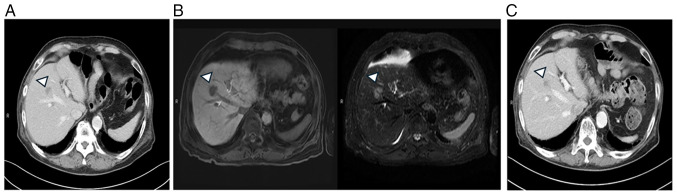
Contrast-enhanced CT scan at 25 months post-operatively, MRI at 27 months post-operatively, and contrast-enhanced CT scan at 31 months post-operatively (following four cycles of trastuzumab plus mFOLFOX6). The white triangles indicate the location of the tumor. (A) Contrast-enhanced CT scan at 25 months post-operatively illustrating a faint 15-mm low-density area in segment 8 of the liver. (B) MRI at 27 months demonstrating a 21x19-mm round lesion in segment 8 of the liver, showing low signal intensity on T1-weighted imaging and mildly high signal on T2-weighted imaging, consistent with liver metastasis from gastric cancer and demonstrated a continued tendency to enlarge. (C) Contrast-enhanced CT scan at 31 months demonstrating shrinkage of the segment 8 liver metastasis to 13 mm. CT, computed tomography.

**Figure 3:**
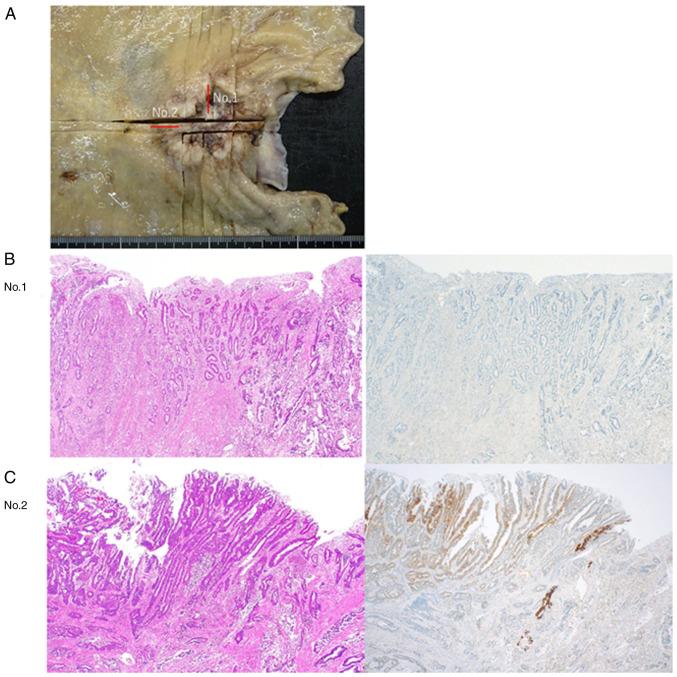
The resected stomach and immunohistochemistry of HER2. (A) The location of the section for the initial examination (No. 1) and for re-evaluation (No. 2). (B) Histological analysis and immunohistochemistry of HER2 at the initial examination. HER2 was not observed in well-differentiated adenocarcinoma (hematoxylin and eosin staining and immunohistochemistry of HER2; magnification, x100). (C) Histological analysis and immunohistochemistry of HER2 at the re-evaluation. Moderate membranous expression of HER2 (score 2+) was noted in the well differentiated adenocarcinoma (hematoxylin and eosin staining and immunohistochemistry of HER2; magnification, x100).

**Figure 4:**
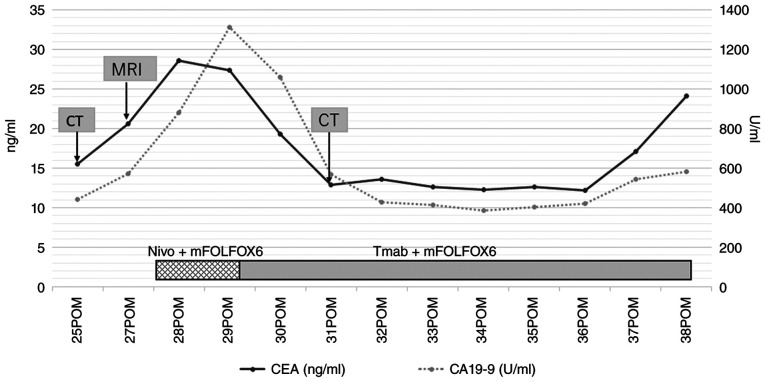
Graph depicting the clinical course of the patient from the onset of liver metastasis through the initiation of chemotherapy, as demonstrated by changes in tumor markers (CEA and CA19-9). The timing of imaging examinations presented in Fig. 2 is also indicated. CEA, carcinoembryonic antigen; CA19-9, carbohydrate antigen 19-9; CT, computed tomography; POM, post-operative month; Nivo, nivolumab; mFOLFOX6, oxaliplatin + leucovorin + 5-fluorouracil regimen; Tmab, trastuzumab.

## Data Availability

The data generated in the present study may be requested from the corresponding author.
